# Effects of early versus delayed application of prone position on ventilation–perfusion mismatch in patients with acute respiratory distress syndrome: a prospective observational study

**DOI:** 10.1186/s13054-023-04749-3

**Published:** 2023-11-27

**Authors:** Xueyan Yuan, Zhanqi Zhao, Yali Chao, Dongyu Chen, Hui Chen, Rui Zhang, Songqiao Liu, Jianfeng Xie, Yi Yang, Haibo Qiu, Leo Heunks, Ling Liu

**Affiliations:** 1 Jiangsu Provincial Key Laboratory of Critical Care Medicine and Department of Critical Care Medicine, Zhongda Hospital, School of Medicine, Southeast University, Nanjing, 210009 Jiangsu China; 2 School of Biomedical Engineering, Guangzhou Medical University, Guangzhou, China; 3 Institute of Technical Medicine, Furtwangen University, Villingen-Schwenningen, Germany; 4 Nanjing Lishui People’s Hospital, Zhongda Hospital Lishui Branch, Southeast University, No. 86 Chongwen Road, Lishui District, Nanjing, 211200 Jiangsu China; 5 Department of Intensive Care, Erasmus University Medical Center, Rotterdam, The Netherlands; 6 Department of Intensive Care, Radboud University Medical Center, Nijmegen, The Netherlands

**Keywords:** Acute respiratory distress syndrome, Prone position, Ventilation/perfusion distribution, Shunt

## Abstract

**Background:**

Prone position has been shown to improve oxygenation and survival in patients with early acute respiratory distress syndrome (ARDS). These beneficial effects are partly mediated by improved ventilation/perfusion (V/Q) distribution. Few studies have investigated the impact of early versus delayed proning on V/Q distribution in patients with ARDS. The aim of this study was to assess the regional ventilation and perfusion distribution in early versus persistent ARDS after prone position.

**Methods:**

This is a prospective, observational study from June 30, 2021, to October 1, 2022 at the medical ICU in Zhongda Hospital, Southeast University. Fifty-seven consecutive adult patients with moderate-to-severe ARDS ventilated in supine and prone position. Electrical impedance tomography was used to study V/Q distribution in the supine position and 12 h after a prone session.

**Results:**

Of the 57 patients, 33 were early ARDS (≤ 7 days) and 24 were persistent ARDS (> 7 days). Oxygenation significantly improved after proning in early ARDS (157 [121, 191] vs. 190 [164, 245] mm Hg, *p* < 0.001), whereas no significant change was found in persistent ARDS patients (168 [136, 232] vs.177 [155, 232] mm Hg, *p* = 0.10). Compared to supine position, prone reduced V/Q mismatch in early ARDS (28.7 [24.6, 35.4] vs. 22.8 [20.0, 26.8] %, *p* < 0.001), but increased V/Q mismatch in persistent ARDS (23.8 [19.8, 28.6] vs. 30.3 [24.5, 33.3] %, *p* = 0.006). In early ARDS, proning significantly reduced shunt in the dorsal region and dead space in the ventral region. In persistent ARDS, proning increased global shunt. A significant correlation was found between duration of ARDS onset to proning and the change in V/Q distribution (*r* = 0.54, *p* < 0.001).

**Conclusions:**

Prone position significantly reduced V/Q mismatch in patients with early ARDS, while it increased V/Q mismatch in persistent ARDS patients.

*Trial registration* ClinicalTrials.gov (NCT05207267, principal investigator Ling Liu, date of registration 2021.08.20).

**Supplementary Information:**

The online version contains supplementary material available at 10.1186/s13054-023-04749-3.

## Background

Prone position has been shown to reduce mortality in patients with early moderate-to-severe acute respiratory distress syndrome (ARDS) [1]. By recruiting dorsal nonaerated but perfused lung tissue and reducing ventral hyperinflation, prone position may improve ventilation/perfusion (V/Q) distribution [2–4] and limit ventilator-induced lung injury (VILI) [5]. In addition, proning has beneficial effects on hemodynamics [6]. Most studies on proning have been conducted in patients with early ARDS, whereas the physiological and clinical effects of proning in persistent ARDS have not been studied in detail. This is however of importance, as it may help clinicians to decide whether proning may be beneficial in patients with persistent ARDS and also help to design future clinical trials [7].

Early ARDS is mainly characterized by exudative inflammation with disruption of the alveolar-capillary barrier, whereas a fibroproliferative phase, characterized by fibroblastic proliferation and organization within the parenchyma, develops later in the course of ARDS [7, 8]. Changes in pulmonary histology in the course of ARDS may affect the response to prone position on V/Q distribution. Therefore, the oxygenation response to prone position may be different between early and persistent ARDS. A recent study provided important insights in the effect of prone position on oxygenation response in COVID-19 ARDS (CARDS) [9]. PaO_2_/FIO_2_ decreased after proning in all patients after 3 weeks of CARDs, whereas such response was unlikely during the first week. In addition, the authors demonstrated that lack of lung recruitment was more frequently in the third week of CARDS, as compared to early CARDS [9]. However, the number of patients recruited was relatively small, and no detailed analysis of V/Q distribution was performed.

Electrical impedance tomography (EIT) is a real-time monitoring technique that is used to assess pulmonary ventilation and perfusion distribution at the bedside [10–12]. The aim of the current study is to compare changes in ventilation and perfusion distribution after proning in early versus persistent ARDS. Given the time-dependent changes in histology, we hypothesize that effects of proning on V/Q distribution are more beneficial in early ARDS.

## Methods

The study was conducted in the medical ICU (Zhongda Hospital, Southeast University, Nanjing, China) and was approved by the Institutional Review Board of Zhongda hospital (No. 2020ZDSYLL057-P01). Informed consent was obtained according to local regulations. This was a prospective, observational cohort sub-study of a study registered in ClinicalTrials.gov (NCT05207267).

### Study population

Consecutive adult patients admitted to our ICU from June 30, 2021, to October 1, 2022, were screened. Inclusion criteria included (1) invasively ventilated; (2) moderate-to-severe ARDS according to the Berlin definition prior to the study [13]; (3) physiological data available before and after prone session. Exclusion criteria were as follows: refusal to participate in the study, contraindications to proning or EIT (e.g., active implantable device, chest malformation, unstable spinal injuries or fractures, and open chest wounds), chronic respiratory diseases that required long-term oxygen therapy, such as pulmonary fibrosis or chronical obstructive pulmonary disease, undrained pneumothorax or pneumomediastinum, and using extracorporeal membrane oxygenation.

### Study protocol and measurements

The following patient characteristics were collected at the enrollment: demographic data (age, gender, Body Mass Index), Sequential Organ Failure Assessment (SOFA), timing of ARDS onset before first prone session, duration of invasive mechanical ventilation (MV), ARDS etiology, disease severity, and ICU mortality. The potential for lung recruitment was assessed by recruitment-to-inflation (R/I) ratio (Additional file 1) [14]. The results of CT scan quantitative analysis were also measured at enrollment (Additional file 1).

All patients were sedated and under neuromuscular blockade during measurements. Throughout the study period, patients were mechanically ventilated in volume-controlled mode and ventilator settings standardized: tidal volume 6–8 ml/kg of predicted body weight, respiratory rate to maintain pH between 7.35 and 7.45, PEEP was set after a recruitment maneuver and a decremental PEEP titration to obtain the highest compliance of respiratory system in supine and unchanged during prone position. FIO_2_ was set to achieve an oxygen saturation (SpO_2_) target of 90–98%. Study procedures were performed after clinical stabilization. EIT measurements, arterial blood gas analyses, respiratory mechanics, and hemodynamic parameters (heart rate, mean arterial pressure) were obtained in supine position before just proning and 12 h after the first prone position session. In our center, prone position was initiated as soon as eligibility criteria were met (Additional file 1).

EIT data were acquired with the PulmoVista 500 (Draeger Medical, Lubeck, Germany) with a sample rate of 50 Hz and stored for offline analysis. The 16-electrode silicon EIT belt was positioned along the fourth to fifth intercostal space and kept in the same position during both supine position and prone position. After the recording of EIT data for 5 min, a 20-s end-expiratory breath hold was performed. Two seconds after the start of occlusion, a bolus of 10% NaCl 10 mL solution was manually injected via central venous catheter [15].

### EIT data analysis

*EIT ventilation maps* were obtained by averaging tidal variation (the difference between end-inspiratory and end-expiratory impedance) over 1-min consecutive breaths. EIT images were analyzed regionally by splitting the tidal variation images into two craniocaudal regions of interest: ventral and dorsal. By analyzing the last minute of each study phase, the following parameters were calculated [15]:

1. Lung ventilated regions, which were defined as the pixels for which tidal variations were ≥ 20% of the maximal pixel impedance variation.
2. Percentage of ventilation distributed in the dorsal regions.
3. Regional ventilation delay (RVD) index [16], which represents the delay between the beginning of inspiration and the culmination of a specific impedance threshold.

*EIT perfusion maps* were obtained from the slope of the impedance decrease following a saline bolus injection during an end-expiratory occlusion. In addition, the following perfusion-related parameters were calculated [15]:

1. Pulmonary perfusion regions, defined as pixels in the perfusion maps with values ≥ 20% of the maximum pixel value.
2. Percentage of perfusion distributed in the dorsal regions.

Subsequently, different regions were identified by combining the ventilation and perfusion regions:

1. Shunt area %: all pixels that were perfused, but not ventilated (pure shunt), divided by the total number of pixels classified as ventilated or perfused.
2. Dead space area %: all pixels that were ventilated, but not perfused (pure dead space), divided by the total number of pixels classified as ventilated or perfused.
3. V/Q mismatch %: the sum of shunt and dead space area.
4. Dead space to shunt ratio: the ratio between dead space area and shunt area.

### Endpoints

The primary endpoint was the difference in change of V/Q distribution between supine and prone position in early versus persistent ARDS. The secondary endpoints included the difference in the fraction of shunt (dorsal, ventral, and global), dead space (dorsal, ventral, and global), percent of dorsal ventilation and perfusion distribution, RVD, respiratory system compliance, driving pressure, PaO_2_/FIO_2_, delta PaCO_2_, and ventilatory ratio between supine and prone in early versus persistent ARDS.

### Classifications

We selected a cut-off of 7 days of ARDS onset time from the initiation of noninvasive (if applicable) or invasive ventilation to the first prone position session for discriminating between early ARDS and persistent ARDS [7]. For the oxygenation response to prone position, patients were classified as prone responders if an increase in the PaO_2_/FIO_2_ > 20% after 2 h of proning [17].

### Statistical analysis

No formal sample size analysis was performed before initiation of the study. However, a sample size analysis was performed while the study was ongoing. Based on previous on the V/Q distribution in ARDS patients [17, 18], we hypothesized a change in V/Q mismatch induced by prone position of 6% might be clinically relevant. A minimum sample size of 56 patients was required to detect a difference of ≥ 6% in V/Q mismatch after proning with a type I error of 0.05 and statistical power of 80%. Fifty-seven patients were finally enrolled to compensate for potential dropouts. No imputation was employed due to all the analyzed cases were completed. Data are expressed as number (percent) for categorical variables and mean (± standard deviation) or median (interquartile range [IQR]) for continuous variables, as appropriate. Normality was tested by the Shapiro–Wilk test. For the comparisons in variations between supine and prone position and differences in paired measurements (prone minus supine) in early and persistent ARDS, Wilcoxon Mann–Whitney test was applied. For the between-group differences of categorical data, Pearson *χ*^2^ test was performed. Spearman regression coefficient was used to assess the association between timing of ARDS onset and change in V/Q mismatch, shunt, and dead space after prone position. A level of *p* < 0.05 (two-tailed) was considered as statistically significant. Statistical analyses were performed using STATA version 19.0 (Statacorp, College Station, TX, USA) and Prism (GraphPad Prism v9.3, La Jolla, CA).

## Results

### Patient characteristics

Fifty-seven patients were enrolled in the study. Baseline characteristics are shown in Table 1. Thirty-three patients were classified as early ARDS and twenty-four were persistent ARDS. Patient flow through the study is presented in Fig. E1 in the Additional file 1. Of 24 patients with persistent ARDS, 23 (95.8%) had moderate ARDS. The daily PaO_2_/FIO_2_ in the patients with persistent ARDS during the 6 days before enrollment and at enrollment and is presented in Fig. E2 in the Additional file 1.

**Table 1:** Clinical characteristic of the study population

Variable	All patients n = 57	Early ARDS n = 33	Persistent ARDS n = 24	*P*
*Patients’ characteristics*				
Age, yr	71 ± 11	72 ± 11	70 ± 11	0.52
Male, n (%)	37 (64.9)	22 (66.7)	15 (62.5)	0.75
Body mass index, kg/m^2^	25.4 ± 2.9	24.9 ± 2.4	26.1 ± 3.4	0.10
Sequential organ failure assessment score	8.4 ± 3.2	7.6 ± 2.7	9.5 ± 3.6	0.02
Timing of ARDS onset before first prone session, d	5 [3, 8]	3 [2, 4]	9 [8, 11]	< 0.001
Use of noninvasive ventilation before intubation, n (%)	36 (63.2)	16 (48.5)	20 (83.3)	0.007
Duration of noninvasive MV before intubation, d	2 (1, 4)	1 (1, 2)	4 (2.5, 6)	< 0.001
Duration of invasive MV before first prone position, d	3 [2, 6]	3 [1, 4]	6 [3.5, 8.5]	< 0.001
Duration of first prone position, h	17 [15, 18]	17 [16, 18]	16.5 [15, 18]	0.68
*Etiology, n (%)*				0.95
COVID-19 pneumonia	26 (45.6)	15 (45.5)	11 (45.8)	
Pneumonia	28 (49.1)	16 (48.5)	12 (50.0)	
Aspiration	3 (5.3)	2 (6.1)	1 (4.2)	
PaO_2_/FIO_2_, mmHg	155 [130, 187]	168 [137, 188]	133 [118, 164]	0.063
R/I ratio	0.29 [0, 0.54]	0.48 [0.22, 0.58]	0 [0, 0.29]	0.002
*Computed tomography scan*				
Total gas volume, ml	2580 [1755, 3115]	2850 [2111, 3184]	2355 [1616, 2895]	0.21
Overinflated volume, ml	175 [70, 306]	108 [31, 226]	188 [117, 316]	0.10
Normally inflated volume, ml	1257 [851, 1503]	1433 [1209, 1753]	1000 [772, 1503]	0.18
Poorly inflated volume, ml	597 [423, 714]	625 [396, 779]	455 [430, 624]	0.48
Collapsed volume, ml	478 [279, 703]	633 [350, 731]	327 [278, 568]	0.07
ICU mortality	9 (15.8)	5 (15.2)	4 (16.7)	0.88

*ARDS* acute respiratory distress syndrome, *MV* mechanical ventilation, *R/I* recruitment-to-inflation

Values are represented as count (percentage) or median (interquartile range)

*p* indicates Mann–Whitney *U* test and Pearson *χ*2 test between change in value between early ARDS and persistent ARDS

### Change in oxygenation, respiratory mechanics, and hemodynamic variables

**Fig. 1:**
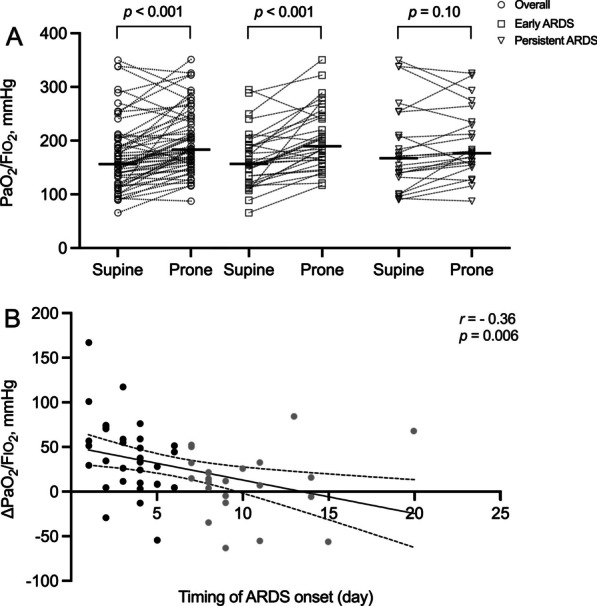
**A** PaO_2_/FIO_2_ changes from supine to prone position for the whole population, patients with early ARDS, and patients with persistent ARDS. **B** The correlation between delta PaO_2_/FIO_2_ and timing of ARDS onset when transitioning to prone position in the patients with early ARDS and persistent ARDS. Transverse line: median. *ARDS* acute respiratory distress syndrome

Table 2 and Fig. 1 present the PaO_2_/FIO_2_ for the whole population and subgroups based on the timing of ARDS onset during the prone session. For the whole population, PaO_2_/FIO_2_ in the prone position was significantly as higher compared to supine position (Fig. 1A). Following proning, oxygenation improved significantly in patients with early ARDS, while it did not change in patients with persistent ARDS (Table 2, Fig. 1A). As shown in Fig. 1B, change in PaO_2_/FIO_2_ between supine and prone position showed a negative correlation with timing of ARDS onset.

**Table 2:** Variations between early and persistent ARDS

Study variables	Early ARDS, *n* = 33		*P*	Persistent ARDS, *n* = 24		*P*
	Supine	Prone		Supine	Prone	
*Ventilator setting*						
FIO_2_	0.4 [0.4, 0.6]	0.4 [0.4, 0.5]	0.01	0.5 [0.4, 0.65]	0.5 [0.4, 0.55]	0.05
Respiratory rate, /min	20 [17, 22]	20 [18, 22]	0.76	27 [20, 30]	26 [20, 30]	0.35
Positive end-expiratory pressure, cmH_2_O	8 [8, 10]	8 [8, 10]	0.26	8 [6, 10]	8 [6, 10]	0.98
Tidal volume, mL/kg predicted body weight	7 [6, 8.1]	7 [6, 8.1]	0.59	6.1 [5.1, 7.0]	6.1 [5.1, 7.0]	0.96
*Respiratory mechanics*						
Plateau pressure, cmH_2_O	20.5 [18.6, 21.7]	19.6 [18.8, 21.0]	0.84	19.3 [16.7, 23.6]	20.6 [17.8, 23.4]	0.26
Driving pressure, cmH_2_O	11.0 [10.3, 12.6]	11.9 [9.4, 13.5]	0.62	12.3 [9.6, 13.6]	12.2 [11.4, 13.4]	0.24
Respiratory system compliance, ml/cmH_2_O	36.0 [31.2, 41.8]	35.9 [29.3, 44.0]	0.30	30.8 [26.8, 38.7]	29. 8 [23.6, 37.0]	0.04
*Arterial blood gases*						
pH	7.40 [7.38, 7.45]	7.41 [7.38, 7.45]	0.54	7.39 [7.37, 7.45]	7.39 [7.35, 7.42]	0.98
PaCO_2_, mmHg	40.1 [37.8, 42.1]	38.3 [35.6, 40.5]	0.02	42.6 [39.7, 44.6]	42.3 [38.2, 52.6]	0.94
PaO_2_, mmHg	72.4 [66.8, 78.4]	82.6 [73.1, 97.8]	< 0.001	84.7 [73.9, 101.8]	84.8 [77.3, 102.9]	0.55
PaO_2_/FIO_2_	157 [121, 191]	190 [164, 245]	< 0.001	168 [136, 232]	177 [155, 232]	0.10
Ventilatory ratio	1.42 [1.24, 1.87]	1.30 [1.21, 1.60]	0.01	1.84 [1.43, 2.11]	1.72 [1.5, 2.06]	0.48
*Electrical impedance tomography*						
V_T_ distribution dorsal, %	36.5 [29.5, 42.6]	60.4 [47.9, 73.8]	< 0.001	36.9 [29.6, 45.7]	57.2 [44.9, 66.0]	0.002
Perfusion distribution dorsal, %	47.9 [42.5, 52.2]	59.1 [54.0, 65.7]	0.001	45.3 [41.1, 50.5]	56.6 [49.5, 64.2]	0.001
COV index	44.8 [41.6, 46.8]	54.6 [49.7, 58.6]	< 0.001	44.3 [40.9, 47.5]	54.6 [49.1, 57.1]	< 0.001
RVD index	6 [4, 8]	4 [4, 5]	< 0.001	6 [4, 7]	5 [4, 6.5]	0.38
Shunt, %	11.4 [7.1, 18.5]	12.7 [6.9, 17.8]	0.21	12.3 [7.4, 20.6]	19.4 [12.6, 24.6]	0.002
Shunt, dorsal, %	8.2 [4.8, 13.8]	3.8 [1.5, 9.6]	0.005	7.7 [3.9, 12.6]	10.7 [6.2, 16.4]	0.04
Shunt, ventral, %	2.0 [0.6, 5.5]	5.7 [2.9, 9.9]	0.02	3.6 [1.4, 8.0]	5.5 [2.9, 11.9]	0.02
Dead space, %	14.2 [7.7, 21.9]	9.1 [5.3, 13.4]	0.004	11.9 [9.4, 16.5]	11.3 [5.9, 15.6]	0.15
Dead space, dorsal, %	3.3 [0.9, 5.5]	3.1 [1.3, 5.2]	0.64	3.7 [1.3, 6.6]	2.9 [0.9, 6.4]	0.16
Dead space, ventral, %	10.0 [5.2, 17.6]	5.9 [2.0, 8.8]	0.001	8.5 [6.5, 10.1]	6.9 [4.9, 10.2]	0.36
Dead space/shunt ratio	1.1 [0.5, 2.9]	0.7 [0.4, 1.7]	0.48	1.2 [0.4, 2.1]	0.6 [0.3, 1.1]	0.009
Dead space/shunt ratio, dorsal	0.4 [0.1, 0.9]	0.7 [0.2, 2.2]	0.22	0.3 [0.1, 1.4]	0.2 [0.1, 0.8]	0.14
Dead space/shunt ratio, ventral	2.0 [0.8, 6.0]	0.3 [0.1, 0.8]	< 0.001	2.0 [0.8, 4.2]	1.1 [0.5, 2.7]	0.07
Total unmatched units, %	28.7 [24.6, 35.4]	22.8 [20.0, 26.8]	< 0.001	26.9 [20.1, 31.4]	31.2 [24.5, 36.4]	0.02
Unmatched units, dorsal, %	13.0 [9.2, 20.6]	9.0 [5.5, 13.2]	< 0.001	12.8 [10.0, 16.3]	17.3 [11.2, 20.2]	0.095
Unmatched units, ventral, %	14.8 [11.3, 18.4]	13.4 [9.4, 14.9]	0.094	12.4 [9.2, 16.9]	14.8 [11.2, 20.2]	0.179
*Hemodynamic variables*						
Heart rate (beats per minute)	80 [69, 90]	81 [72, 95]	0.50	85 [69, 93]	85 [75, 93]	0.54
MAP (mmHg)	82 [74, 88]	82 [75, 90]	0.28	83 [68, 87]	74 [69, 87]	0.80

*ARDS* acute respiratory distress syndrome, *COV* center of ventilation, *RVD* regional ventilation delay index, *SBP* systolic blood pressure, *MAP* mean arterial pressure

Values are represented as median (interquartile range)

*p* indicates Wilcoxon signed-rank tests between paired supine position and prone position values

The change in the respiratory mechanics and hemodynamic variables after proning during the first session is described in Table 2.

### Change in ventilation and perfusion

With proning, ventilation and perfusion changed as described in Table 2. The percentage of *V*_T_ distribution to the dorsal regions increased for the whole population, in patients with early ARDS, and in persistent ARDS. Similarly, proning caused significant increase in perfusion distribution to the dorsal region for the whole population, patients with early ARDS, and persistent ARDS. Distribution of ventilation and perfusion to the dorsal lung regions were not significantly associated with timing of ARDS onset (Fig. E3 in the Additional file 1).

### Change in V/Q distribution

**Fig. 2:**
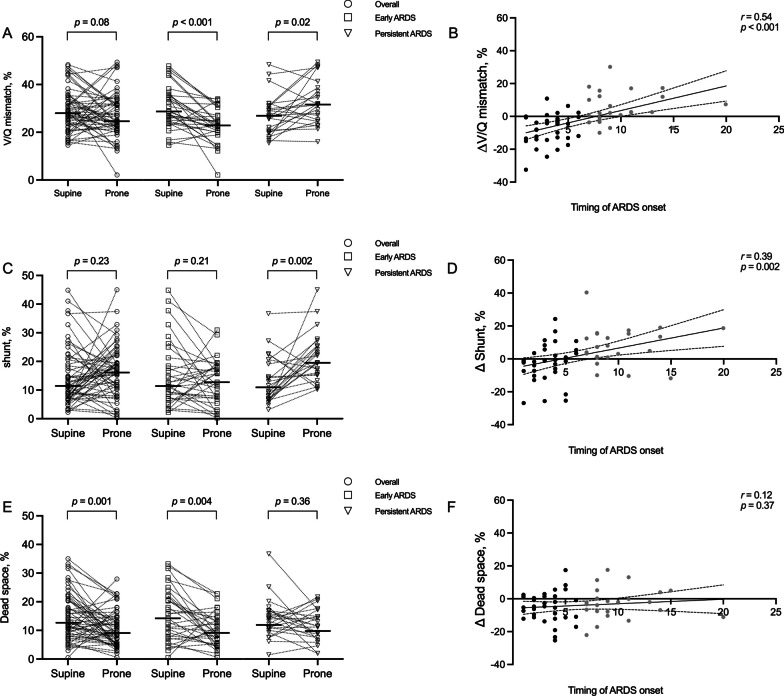
Changes from supine to prone position for the whole population, patients with early ARDS, and patients with persistent ARDS in V/Q mismatch (**A**), shunt (**C**), and dead space (**E**) at the global. Correlation between timing of ARDS onset with difference in ventilation–perfusion mismatch (**B**), shunt (**D**), and dead space (**F**) at the global. Transverse line: median. Each dot represents one patient: dark gray dots denote patients with early ARDS and light gray dots denote patients with persistent ARDS. The dotted line represents 95% confidence intervals, while the solid line represents regression line. *V/Q* ventilation–perfusion, *ARDS* acute respiratory distress syndrome

Changes in proning-induced V/Q distribution were different in early ARDS versus persistent ARDS (Fig. 2A). In patients with early ARDS, prone position significantly decreased V/Q mismatch (*p* < 0.001), while prone position increased V/Q mismatch (*p* = 0.02) in patients with persistent ARDS.

The fraction of shunt in the supine and prone for the whole population and subgroups is presented in Fig. 2C. In patients with early ARDS, global shunt was not significantly different between supine and prone position. Furthermore, shunt significantly decreased in the dorsal region (*p* = 0.005) and increased in the ventral region (*p* = 0.02) after proning. In patients with persistent ARDS, global, dorsal, and ventral region shunt significantly increased after proning (Table 2).

The fraction of dead space at the two body positions is presented in Fig. 2E. For the whole population, global dead space decreased (*p* = 0.001), and decreased also in the ventral region (*p* = 0.001) after proning, whereas dead space in the dorsal region was not significantly different after proning. In contrast to the persistent ARDS subgroup, dead space decreased in the global (*p* = 0.004), and ventral region (*p* = 0.001, Table 2) in patients with early ARDS.

**Fig. 3:**
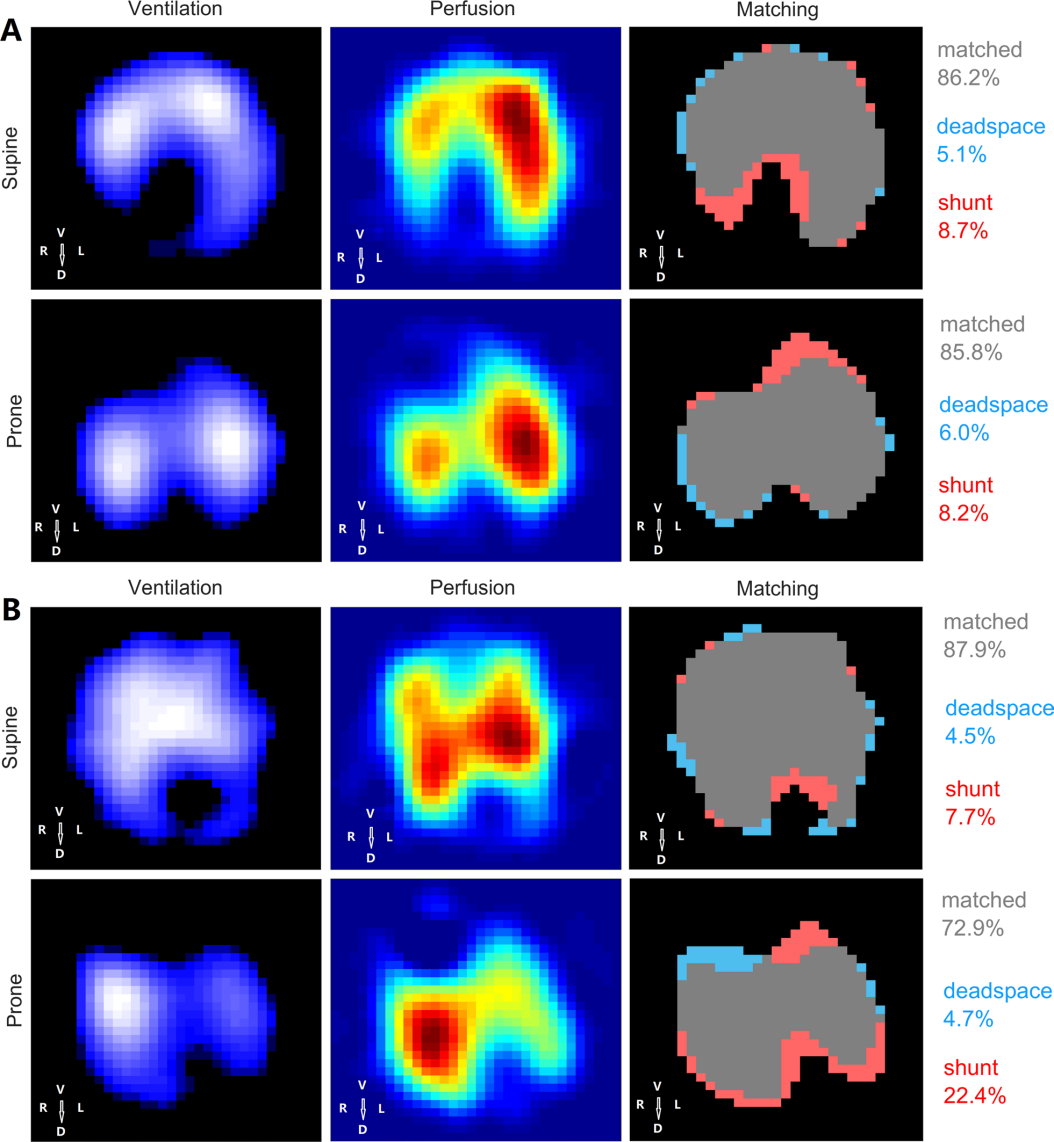
Effect of prone position on ventilation/perfusion (V/Q) matching in two representative patients. From left to right, lung ventilation (blue–white gradient area), perfusion (red–yellow area), and V/Q matching are depicted. First row, supine position. Second row, prone position. First column, functional EIT image shows tidal ventilation distribution (blue–white gradient area). Second column, functional EIT image shows perfusion distribution (red–yellow area). Third column, functional EIT image shows the distribution of regional V/Q matching. Ventilated regions were defined as pixels with impedance changes higher than 20% of the maximum tidal impedance variation in the functional ventilation image. Perfused regions were defined as pixels higher than 20% of the maximum bolus-related impedance change in the functional perfusion image. Regions with high ventilation and low perfusion are marked in blue (denoted as dead space), low ventilation and high-perfusion regions in red (denoted as shunt), and good V/Q matching in gray (denoted as V/Q matching). The first patient with early ARDS **A** showed decreased percent of dorsal shunt and ventral dead space units, leading to decreased mismatch. The second patient with persistent ARDS **B** showed decreased percent of total shunt, leading to increased mismatch

The physiologic effects of prone position based on the pulmonary ventilation and perfusion in two representative patients, monitoring by EIT, are presented in Fig. 3.

### The factors predicting the efficacy of prone position on the V/Q matching

For the effect of time between onset of ARDS and the studied prone session on V/Q distribution, shunt and dead space is shown in Fig. 2B, D, F.

### Oxygenation responders versus non-responders

Table E1 in the Additional file 1 shows patient characteristics of the responders and non-responders based on PaO_2_/FIO_2_. For the oxygenation response to proning, 26 patients were classified as responders. The responders had the shorter timing of ARDS onset before first prone session and higher R/I index when compared to non-responders (*p* = 0.011 and *p* = 0.026, respectively).

## Discussion

This prospective clinical study was designed to investigate the effects of prone position on gas exchange and respiratory mechanics in patients with early versus persistent ARDS. We demonstrate that the effects of prone position on gas exchange depends on the timing since ARDS onset. To summarize, (1) prone position decreased V/Q mismatch in early ARDS, but increased V/Q mismatch in persistent ARDS; (2) proning increased shunt in patients with persistent ARDS, without affecting shunt in early ARDS (3) proning decreased dead space in early ARDS, but not in persistent ARDS; and finally (4) proning improved oxygenation in early ARDS, but not in persistent ARDS.

### Lung recruitment in early versus persistent ARDS

In early ARDS, lung edema, mediastinal weight, and intra-abdominal pressure may contribute to increased ventral-dorsal pleural pressure gradient, facilitating development of atelectasis and such reduce ventilation in the dependent lung regions [19]. In transitioning to prone position, the pleural pressure gradient from nondependent to dependent regions is reduced [20]. As a result, dorsal alveoli may be recruited improving ventilation. Indeed, a previous study using CT scanning in supine and prone position demonstrated dorsal lung recruitment after proning in unselected ARDS patients [5].

In persistent ARDS, prone position may decrease alveolar overdistension in the ventral region due to the decrease of chest wall compliance, and further facilitate distribution of ventilation from ventral to dorsal region. In a pilot study, Xin et al. evaluated the change in regional ventilation between supine and prone position in persistent ARDS (*N* = 2), and they found EIT-measured compliance did not improve in the dorsal region but worsened in the ventral region after proning, suggesting reduced lung distension in the ventral region after proning [21]. In the current study, evaluating many more patients, we found that both in early and persistent ARDS, a significant shift in tidal volume distribution from ventral to dorsal lung regions occurred after transition to prone position.

### Lung perfusion in early versus persistent ARDS

Pulmonary blood flow to different portions of the lung is regulated by several physiological mechanisms, including airspace compression of vessels, lung/heart geometry, and hypoxic pulmonary vasoconstriction [2]. On the other hand, gravity has limited impact on local pulmonary perfusion. Hence, a lack of perfusion redistribution has been demonstrated in patients with early ARDS after prone position [17, 22, 23]. This is in apparent contrast with our study, showing that the percentage of pulmonary dorsal perfusion significantly increased both in early and persistent ARDS, when turning from supine to prone position. Monitoring pulmonary perfusion in our study was however performed at 12 h after prone position initiation, which may help to explain the apparent discrepancy. Indeed, Wang et al. showed that compared to supine position, pulmonary dorsal perfusion was significantly increased after an average of 15.5 h in prone position [18]. The reduction in hypoxic vasoconstriction, less heart superimposed pressure, and less airspace compression of vessels in the dorsal region, to some extent, may contribute to the increase in dorsal perfusion after proning. Furthermore, a previous study reported marked interindividual variability in dorsal perfusion response after proning [24]. Hence, individualized dynamic lung perfusion monitoring may be warranted to assess the perfusion response of positional interventions.

### V/Q distribution in early versus persistent ARDS

Although it is well-known that oxygenation improves in hypoxemic patients in prone position, we found this is restricted to patients with early ARDS, while proning did not affect oxygenation in patients with persistent ARDS. This may be explained by the difference in effect of prone position on V/Q matching in these two groups. Previous studies showed that an improvement in the V/Q matching, rather than lung recruitment per se, explains improved oxygenation after prone position in the early ARDS [17, 18]. Consistently, our study demonstrates the increase in V/Q matching after prone position was restricted to patients with early ARDS, while V/Q distribution deteriorated in persistent ARDS following prone position. Accordingly, timing since ARDS onset appears an important factor in the effect of proning on V/Q distribution. This may be explained by reduced ability of alveolar recruitment and increased distributing of perfusion in the dorsal regions in persistent ARDS.

The observation that global shunt in patients with early ARDS was unmodified after proning is consistent with previous observations in patients with CARDS [17, 23]. Following prone position, functional stiffening of chest wall leads to a decrease in the total chest wall compliance. Consequently, ventilation in the ventral region decreased, resulting in the increase in ventral shunt in these patients. In patients with persistent ARDS, shunt significantly increased both in the ventral and dorsal regions. For this group of patients, low lung recruitability was confirmed with lower R/I ratio [14]. It is possible that prone position may merely inflate the aerated alveoli, rather than recruit nonaerated alveoli in the dorsal region due to low lung recruitability. Minimal alveolar recruitment, with increased perfusion, resulted in an increase in dorsal shunt. This finding is consistent with evolution toward a “fibrosis-like” pattern in lung pathology [9]. In our study, unmodified shunt and improved oxygenation after prone position in patients with early ARDS is consistent with previous studies [17, 23]. Of note, EIT can only identify pure shunt, which differs from “physiological shunt” as calculated by Berggren shunt equation in that it excludes the contribution from low V/Q areas [25]. Hence, we hypothesize that improved oxygenation after proning can be explained by reduction in regions with low V/Q ratio, as previously described [23].

Prone position decreased the ventral dead space since it decreased ventilation in this region which tended to cause alveolar overdistension in patients with early ARDS. For patients with persistent ARDS, dead space did not alter despite of the potential collapse of ventral region. Interestingly, PaCO_2_ and ventilatory ratio were significantly reduced when turning from supine to prone position in the early ARDS, but not in the persistent ARDS. This discrepancy may be explained by the marked decrease in ventral perfusion after proning in patients with persistent ARDS.

This study has some limitations: (1) Considering the effect of prone position on the chest wall elastance and lung elastance, esophageal pressure can further determine the change of lung compliance between supine position and prone position. However, these data were not obtained in our study. (2) EIT cannot provide images of the whole lung, and it only provides a validated projection of a three-dimensional distribution of ventilation and perfusion on a two-dimensional axial plane, so it is not enough for assessment of the entire spectrum of V/Q matching in the lung. (3) Due to lack of cardiac output, pixel-level V/Q ratios measured by EIT were relative. Additionally, the percentage of low V/Q units and high V/Q units was not obtained. (4) The method for EIT perfusion requires further clinical validation and we did not directly assess the effects of proning on hemodynamics. (5) Our finding regarding V/Q distribution were derived from EIT acquisition. The results should be generalized with caution due to different principles of different techniques used for monitoring V/Q distribution. (6) Patients classified as “persistent ARDS” may be comprise a rather heterogeneous of patients. This cohort may include patients with slowly worsening ARDS and patients that already met indication for prone position at an earlier time point. However, proning was not standard of care in some of the center referring patients to our center.

### Clinical implications

This study shows that the effects of prone position on oxygenation are limited in persistent ARDS, in contrast to early ARDS. In line with an original study and a recent guideline, prone position should be initiated early in the course of ARDS [1, 26]. Worth mentioning, the benefit of prone position in improved survival is not attributed to improved oxygenation [27, 28]. However, VILI was not assessed in this study. Hence, despite the limited response of proning on oxygenation and V/Q matching in persistent ARDS, the effect on mortality in these patients remains to be investigated.

## Conclusions

In conclusion, timing from ARDS onset is an important factor in the effect of proning on oxygenation response in patients with ARDS. Proning at a later stage of ARDS has limited effect on oxygenation. However, it is important to recognize the beneficial effects of proning in previous studies were not explained by the oxygenation response.

### Supplementary Information

**Additional file 1**. **Table E1.** change of variations between oxygenation responders and non-responders. **Figure E1.** The flow chart of patients through study. **Figure E2.** PaO2/FIO2 in the patients with persistent ARDS during the 6 days before enrollment and at enrollment. **Figure E3.** **A** Correlation between timing of ARDS onset with difference in the percent of ventilation to the dorsal region. **B** Correlation between timing of ARDS onset with difference in the percent of perfusion to the dorsal region.

## Data Availability

The datasets used and/or analyzed during this study are not publicly available but are available from the corresponding authors on reasonable request.

## Abbreviations

ARDS: Acute respiratory distress syndrome
V/Q: Ventilation/perfusion
EIT: Electrical impedance tomography
SOFA: Sequential organ failure assessment
MV: Mechanical ventilation
R/I: Recruitment-to-inflation ratio
SpO_2_: Oxygen saturation
RVD: Regional ventilation delay
