# Supplementary material for: Effects of early versus delayed application of prone position on ventilation–perfusion mismatch in patients with acute respiratory distress syndrome: a prospective observational study
Source: Crit Care. 2023 Nov 27;27:462. doi: 10.1186/s13054-023-04749-3 (PMC10683149; doi:10.1186/s13054-023-04749-3)
Supplement: Supplementary file 1 — Additional file 1. Table E1. change of variations between oxygenation responders and non-responders. Figure E1. The flow chart of patients through study. Figure E2. PaO2/FIO2 in the patients with persistent ARDS during the 6 days before enrollment and at enrollment. Figure E3. A Correlation between timing of ARDS onset with difference in the percent of ventilation to the dorsal region. B Correlation between timing of ARDS onset with difference in the percent of perfusion to the dorsal region. [file 13054_2023_4749_MOESM1_ESM.docx]

**Supplemental digital content**

**Effects of early and delayed application of prone position on ventilation-perfusion mismatch in patients with acute respiratory distress syndrome: A Prospective Observational Study**

Xueyan Yuan, M.D., Zhanqi Zhao, Ph.D., Yali Chao, M.D., Dongyu Chen, M.D., Hui Chen, M.D., Ph.D., Rui Zhang, M.D., Songqiao Liu, M.D., Ph.D., Jianfeng Xie, M.D., Ph.D., Yi Yang, M.D., Ph.D., Haibo Qiu^#^, M.D., Ph.D, Leo Heunks^#^, Ph.D., Ling Liu^#^, M.D., Ph.D.

Xueyan Yuan, Jiangsu Provincial Key Laboratory of Critical Care Medicine and Department of Critical Care Medicine, Zhongda Hospital, School of Medicine, Southeast University, Nanjing, 210009, Jiangsu, China. [18826401594@163.com](mailto:18826401594@163.com)

Zhanqi Zhao, School of Biomedical Engineering, Guangzhou Medical University, Guangzhou, China and Institute of Technical Medicine, Furtwangen University, Villingen-Schwenningen, Germany. zhanqi.zhao@hs-furtwangen.de

Yali Chao, Jiangsu Provincial Key Laboratory of Critical Care Medicine and Department of Critical Care Medicine, Zhongda Hospital, School of Medicine, Southeast University, Nanjing, 210009, Jiangsu, China. [chaoyali@126.com](mailto:chaoyali@126.com)

Dongyu Chen, Jiangsu Provincial Key Laboratory of Critical Care Medicine and Department of Critical Care Medicine, Zhongda Hospital, School of Medicine, Southeast University, Nanjing, 210009, Jiangsu, China. [15366103007@163.com](mailto:15366103007@163.com)

Hui Chen, Jiangsu Provincial Key Laboratory of Critical Care Medicine and Department of Critical Care Medicine, Zhongda Hospital, School of Medicine, Southeast University, Nanjing, 210009, Jiangsu, China. 15905162429@163.com

Rui Zhang, Jiangsu Provincial Key Laboratory of Critical Care Medicine and Department of Critical Care Medicine, Zhongda Hospital, School of Medicine, Southeast University, Nanjing, 210009, Jiangsu, China. zr1660919338@163.com

Songqiao Liu, Jiangsu Provincial Key Laboratory of Critical Care Medicine and Department of Critical Care Medicine, Zhongda Hospital, School of Medicine, Southeast University, Nanjing, 210009, Jiangsu, China and Nanjing Lishui People's Hospital, Zhongda Hospital Lishui Branch, Southeast University, No. 86 Chongwen Road, Lishui District, Nanjing, 211200, Jiangsu, China. liusongqiao@ymail.com

Jianfeng Xie, Jiangsu Provincial Key Laboratory of Critical Care Medicine and Department of Critical Care Medicine, Zhongda Hospital, School of Medicine, Southeast University, Nanjing, 210009, Jiangsu, China. [xie820405@126.com](mailto:xie820405@126.com)

Yi Yang, Jiangsu Provincial Key Laboratory of Critical Care Medicine and Department of Critical Care Medicine, Zhongda Hospital, School of Medicine, Southeast University, Nanjing, 210009, Jiangsu, China. [yiyiyang2004@163.com](mailto:yiyiyang2004@163.com)

Haibo Qiu, Jiangsu Provincial Key Laboratory of Critical Care Medicine and Department of Critical Care Medicine, Zhongda Hospital, School of Medicine, Southeast University, Nanjing, 210009, Jiangsu, China. [haiboq2000@163.com](mailto:haiboq2000@163.com)

Leo Heunks, Department of Intensive Care, Erasmus University Medical Center, Rotterdam, the Netherlands. l.heunks@erasmusmc.nl

Ling Liu, Jiangsu Provincial Key Laboratory of Critical Care Medicine and Department of Critical Care Medicine, Zhongda Hospital, School of Medicine, Southeast University, Nanjing, 210009, Jiangsu, China. liulingdoctor@126.com

**Methods**

**Study protocol**

Patients were deeply sedated, paralyzed, and mechanically ventilated on volume-controlled mode. Ventilator settings, prone position (PP), and electrical impedance tomography (EIT) measurements were all standardized for all patients during the study period as follows.

**Ventilator Settings**: FIO_2_ was set to achieve an oxygen saturation (SpO_2_) target of 90-98%. Respiratory rate (RR) was set to maintain pH between 7.35 and 7.45. Tidal volume 6-8ml/kg of predicted body weight, positive end-expiratory pressure (PEEP) was set after a recruitment maneuver and a decremental PEEP titration to obtain the highest compliance of respiratory system in supine. Both tidal volume and PEEP remained unchanged throughout the study period, under the premise of lung protective ventilation and patients’ condition permitting.

**Prone Position**: The decision to initiate PP and the timing were at the discretion of the attending clinician. However, PP was recommended in patients with PaO_2_/FIO_2_ < 150 mm Hg and FIO_2_ ≥ 60%, despite optimization of PEEP (E1). The length of PP sessions will be at least of 12 h/day (E2). The criteria for stopping PP included the development of complications, i.e. extubation, displacement of endotracheal tube (ET) to mainstem bronchus, ET obstruction, hemoptysis, SpO_2_ < 85% or PaO_2_ < 55 mmHg for > 5 min despite FIO_2_ at 100%, bradycardia < 30 bpm for >1 min, mean arterial pressure < 60 mmHg for >5 min, cardiac arrest.

**Electrical Impedance Tomography measurements**: The 16-electrode silicon EIT belt was positioned along the fourth to fifth intercostal space, and kept in the same position during both supine position and PP. EIT data were acquired with the PulmoVista 500 (Draeger Medical, Lubeck, Germany) with a sample rate of 50 Hz, and stored for offline analysis.

Patients who didn’t receive PP before enrollment and scheduled for PP upon clinical decision of the attending physicians were enrollment. Subsequently, the EIT measurements, arterial blood gas analyses, respiratory mechanics, and hemodynamic parameters (heart rate, mean arterial pressure) were obtained in the supine position (baseline). 12 hours after PP in the first session, these measurements were again obtained within the target state.

**Gas exchange data computation**

Ventilatory Ratio (VR) was computed as (minute ventilation * PaCO2)/(PBW*37.5*100) (E3).

**Respiratory mechanics data computation**

The measurement of respiratory mechanics was conducted by tele-inspiratory and tele-expiratory occlusions of 2-3 seconds, under the condition of volume-controlled mode and no spontaneous breathing effort. Driving pressure (DP) was calculated as the difference between plateau pressure (Pplat) and total positive end-expiratory pressure (PEEP). Respiratory system compliance (Crs) was calculated as the ratio between tidal volume and DP.

**Recruitment-to-inflation ratio**

Recruitment-to-inflation (R/I) ratio, a simple accessible and reliable method to assess lung recruitability at the bedside, was recently developed to differentiate recruiters and non-recruiters (E4). Considering its misinterpretation of respiratory mechanics data, presence of complete airway closure was assessed by measuring airway opening pressure (AOP) during a low flow insufflation (5 L/min), as described previously. The R/I ratio was determined with high PEEP and low PEEP set at 15 cm H_2_O and 5 cm H_2_O (or AOP, either of which was higher), respectively. During a single breath, change in the end-expiratory lung volume (ΔEELV) was measured by releasing high PEEP to low PEEP, and the recruited volume (ΔVrec) was calculated as the difference between the measured ΔEELV and the predicted ΔEELV. ΔVrec divided by the effective pressure change gave the recruited lung’s compliance (Crec). Finially, the R/I ratio was defined as the ratio between Crec and Crs at the low PEEP.

**CT analysis**

All CT-scan imaging was obtained and contoured by 2 independent researchers and analyzed offline, using an open-source software (3D Slicer). The global lung volume and the proportions of hyperinflated, normally-, poorly- and non-aerated lung volume were computed by quantitative analysis, as previously described (E5).

**References**

1. Guérin C, Reignier J, Richard JC, Beuret P, Gacouin A, Boulain T, et al; PROSEVA Study Group. Prone positioning in severe acute respiratory distress syndrome. N Engl J Med. 2013;368:2159-68.

2. Fan E, Del Sorbo L, Goligher EC, Hodgson CL, Munshi L, Walkey AJ, et al; American Thoracic Society, European Society of Intensive Care Medicine, and Society of Critical Care Medicine: American Thoracic Society, European Society of Intensive Care Medicine, and Society of Critical Care Medicine. An official American Thoracic Society/European Society of Intensive Care Medicine/Society of Critical Care Medicine clinical practice guideline: Mechanical ventilation in adult patients with acute respiratory distress syndrome. Am J Respir Crit Care Med. 2017;195:1253-63.

3. Sinha P, Calfee CS, Beitler JR, Soni N, Ho K, Matthay MA, et al. Physiologic analysis and clinical performance of the ventilatory ratio in acute respiratory distress syndrome. Am J Respir Crit Care Med. 2019;199:333-41.

4. Chen L, Del Sorbo L, Grieco DL, Junhasavasdikul D, Rittayamai N, Soliman I, et al. Potential for lung recruitment estimated by the recruitment-toinflation ratio in acute respiratory distress syndrome. A clinical trial. Am J Respir Crit Care Med. 2020;201:178-87.

5. Gattinoni L, Caironi P, Cressoni M, Chiumello D, Ranieri VM, Quintel M, et al. Lung recruitment in patients with the acute respiratory distress syndrome. N Engl J Med. 2006;354:1775-86.

**Table E1. change of variations between oxygenation responders and non-responders**

| Study variables | Responders  n = 26 | Non-responders  n = 31 | *P* |
| --- | --- | --- | --- |
| Patients’ characteristics | | | |
| Age, yr | 72 ± 11 | 70 ± 10 | 0.588 |
| Male, n (%) | 18 (69.2) | 19 (61.3) | 0.532 |
| Body mass index, kg/m^2^ | 24.4 [23.4, 26.7] | 24.7 [22.9, 26.5] | 0.885 |
| Sequential Organ Failure  Assessment score | 9 [8, 10] | 7 [6, 10] | 0.068 |
| Timing of ARDS onset before first prone session, d | 4 [2, 7] | 8 [4, 9] | 0.011 |
| Use of noninvasive ventilation before intubation, n (%) | 16 (61.5) | 20 (64.5) | 0.816 |
| Duration of noninvasive MV before intubation, d | 1.0 [1.0, 3.0] | 3.5 [1.8, 6.0] | 0.025 |
| Duration of first prone position, h | 17.0 [15.0, 18.0] | 17.5 [15.5, 18.0] | 0.301 |
| Etiology, n (%) | | | 0.127 |
| COVID-19 pneumonia | 10 (38.5) | 16 (51.6) |  |
| Pneumonia | 13 (50.0) | 15 (48.4) |  |
| Aspiration | 3 (11.5) | 0 |  |
| PaO_2_/FIO_2_, mmHg | 148 [118, 178] | 168 [134, 189] | 0.073 |
| R/I ratio | 0.40 [0.15, 0.68] | 0.22 [0, 0.44] | 0.026 |
| Computed tomography scan | | | |
| Total gas volume, ml | 2545 [2212, 3115] | 2850 [1616, 3253] | 0.821 |
| Overinflated volume, ml | 175 [70, 226] | 175 [100, 316] | 0.486 |
| Normally inflated volume, ml | 1257 [963, 1503] | 1372 [851, 2003] | 0.955 |
| Poorly inflated volume, ml | 621 [434, 671] | 483 [399, 843] | 0.624 |
| Collapsed volume, ml | 478 [278, 703] | 523 [285, 683] | 0.821 |
| ICU mortality | 3 (11.5) | 6 (19.4) | 0.420 |

ARDS = acute respiratory distress syndrome, MV = mechanical ventilation, PP = prone position.

Values are represented as count (percentage) or median (interquartile range).

*p* indicates Mann-Whitney *U* test and Pearson χ2 test between change in value between early ARDS and persistent ARDS.

**Figure E1.** The flow chart of patients through study

**Figure E2.** PaO_2_/FIO_2_ in the patients with persistent ARDS during the 6 days before enrollment and at enrollment. D0, day at enrollment. D1-D6, day before enrollment. Each circle represents one patient. The dotted line represents median. The horizontal line of the “T” and reversed “T” represents 95% percentiles and 5% percentiles, respectively. ARDS = acute respiratory distress syndrome. * *p* < 0.05, ** *p* < 0.01.

**
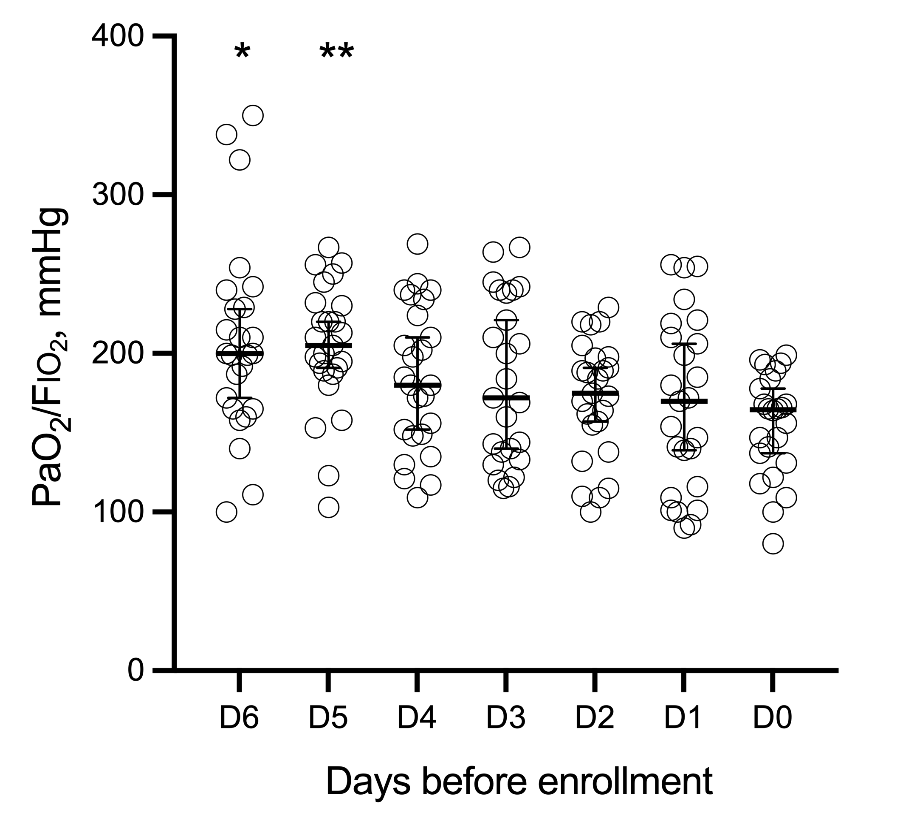
**

**Figure E3.** (A) Correlation between timing of ARDS onset with difference in the percent of ventilation to the dorsal region. (B) Correlation between timing of ARDS onset with difference in the percent of perfusion to the dorsal region. The dotted line represents 95% confidence intervals, while the solid line represents regression line. ARDS = acute respiratory distress syndrome.

**
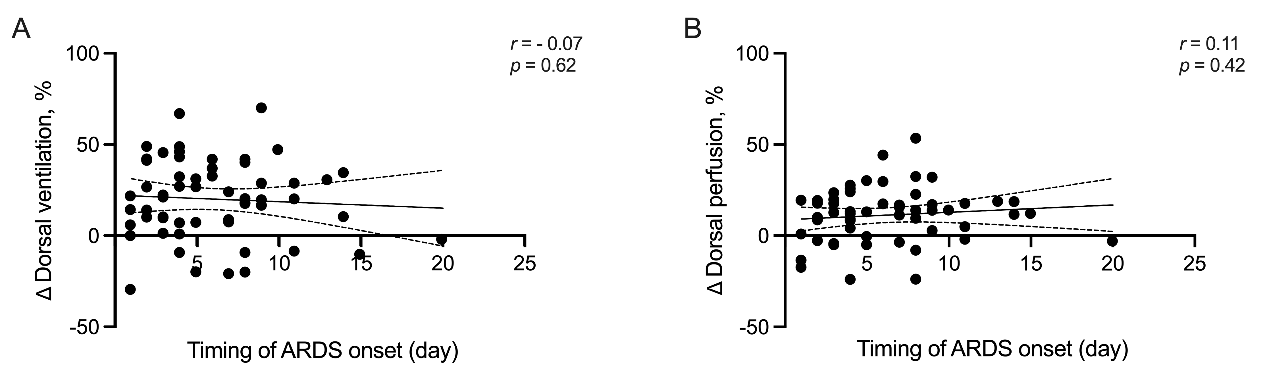
**
